# Artificial Intelligence‐Derived Intramuscular Adipose Tissue Assessment Predicts Perineal Wound Complications Following Abdominoperineal Resection

**DOI:** 10.1002/wjs.70095

**Published:** 2025-09-15

**Authors:** Alex Besson, Ke Cao, Rory Kokelaar, Emina Hajdarevic, Lara Wirth, Josephine Yeung, Justin M. Yeung

**Affiliations:** ^1^ Department of Surgery, Western Precinct The University of Melbourne Melbourne Australia; ^2^ Melbourne Academic Centre for Health North Melbourne Australia; ^3^ Department of Colorectal Surgery Footscray Hospital Western Health Melbourne Australia

**Keywords:** artificial intelligence, body composition, proctectomy, rectal neoplasm

## Abstract

**Background:**

Perineal wound complications following abdominoperineal resection (APR) significantly impacts patient morbidity. Despite various closure techniques, no method has proven superior. Body composition is a key factor influencing postoperative outcomes. AI‐assisted CT scan analysis is an accurate and efficient approach to assessing body composition. This study aimed to evaluate whether body composition characteristics can predict perineal wound complications following APR.

**Methods:**

A retrospective cohort study of APR patients from 2012 to 2024 was conducted, comparing primary closure and inferior gluteal artery myocutaneous (IGAM) flap closure outcomes. Preoperative CT scans were analyzed using a validated AI model to measure lumbosacral skeletal muscle (SM), intramuscular adipose tissue (IMAT), visceral adipose tissue, and subcutaneous adipose tissue.

**Results:**

Greater IMAT volume correlated with increased wound dehiscence in males undergoing IGAM closure (40% vs. 4.8% and *p* = 0.027). Lower SM‐to‐IMAT volume ratio was associated with higher wound infection rates (60% vs. 19% and *p* = 0.04). Closure technique did not significantly impact wound infection or dehiscence rates.

**Conclusion:**

This study is the first to use AI derived 3D body composition analysis to assess perineal wound complications after APR. IMAT volume significantly influences wound healing in male patients having IGAM reconstruction.

## Graphical abstract

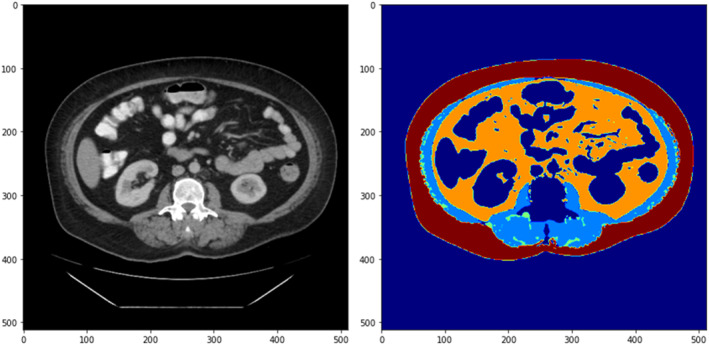

## 1 Introduction

Perineal wound complications following abdominoperineal resection (APR) are a problematic and can occur in up to 65% of patients, causing significant patient morbidity and reduced quality of life [1]. Several methods of perineal closure are used in surgical practice with no consensus in the literature on the superior technique [2]. Individual patient characteristics, neoadjuvant radiotherapy, defect size, and extralevator excision are factors that help to determine the appropriate method of perineal closure [3, 4]. Currently, there is no predictive tool available that can help identify which patients are at greatest risk of wound dehiscence and failure.

Body composition has been increasingly shown to be associated with surgical outcomes [5]. Bimurzayeva et al. showed visceral adiposity is associated with increased postoperative complications (27.1% vs. 19.0% and *p* = 0.028) whereas greater abdominal skeletal muscle volume results in longer overall survival (*p* < 0.001) [5]. Sarcopenia, myosteatosis, and sarcopenic obesity are all key body composition subtypes that have consistently been shown to increase the risk of surgical complications [5, 6, 7, 8]. Two‐dimensional body composition assessment derived from a single computed tomography (CT) slice at the mid L3 vertebrae is historically the gold standard method of assessment [9, 10]. However, the development of artificial intelligence (AI) derived body composition analysis has allowed for the emergence of automated 3D tissue segmentation. This has transformed body composition assessment from a labor‐intensive to an automated, validated, time efficient, and reproducible process [11, 12]. Three‐dimensional analysis is a more accurate assessment of body composition and has greater predictive power for surgical complications and overall survival when compared to 2D or anthropometric analysis [5, 12].

Since the emergence of 3D body composition analysis, there has been significant heterogeneity within the literature regarding how this analysis is assessed. Recent publications in colorectal cancer (CRC) cohorts have looked at multiple slices across the L3 vertebrae, lumbar (L1‐5) vertebral levels, and all lumbosacral (L1‐S5) vertebral levels [5, 13]. The current pitfall of 3D volumetric segmentation is that there are no validated volumetric cutoff thresholds to denote different body composition subtypes [14]. Consequentially, there is currently no accepted value for lumbosacral tissue volume to define sex specific sarcopenia, obesity, or myosteatosis.

The aim of this study was to determine if CT derived 3D body composition characteristics are predictive of perineal wound complications following APR. Additionally, we aim to identify if these findings are influenced by the method of perineal closure, inferior gluteal artery myocutaneous (IGAM) flap, or primary closure (PC). Early identification of at risk patients could allow for patient intervention during a long neoadjuvant treatment period to minimize their risk profile for postoperative complications via nutrition and prehabilitation interventions.

## 2 Materials and Methods

A retrospective study was conducted from January 2012 to May 2024 looking at outcomes following APR with either IGAM flap reconstruction or PC of the perineal wound. Ethics approval was granted by Western Health (WH) Ethics Committee (Project number QA.2018.74). Data and results were reported in accordance with the STROBE checklist.

### 2.1 Data Collection

Patients undergoing APR were identified from the Australian Comprehensive Cancer Outcomes and Research Database (ACCORD), a prospective registry for CRC patients within Victoria, Australia. Patient demographics and surgical outcome data were collected from this database and cross referenced with WH electronic medical records. Wound dehiscence was defined as either superficial or deep dehiscence requiring return to theater or ward based wound management; whereas wound infection required the prescription of antibiotics and clinical or radiological evidence of a postoperative wound infection. All patients in this study received neoadjuvant radiotherapy (50–54 Gray) for rectal adenocarcinoma or anal squamous cell carcinoma (SCC). Since April 2020, patients with locally advanced rectal cancer at WH were considered for total neoadjuvant therapy (TNT). Staging CT scans were collected for all patients with digital imaging and communications in medicine files being downloaded and used for body composition analysis with an in‐house AI software.

### 2.2 Body Composition Measurement

**FIGURE 1:**
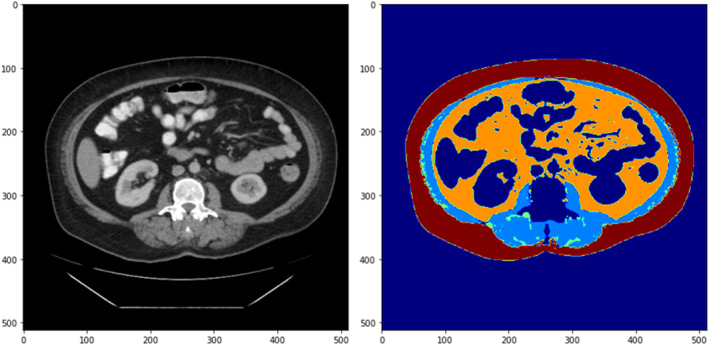
AI mediated body composition analysis. Skeletal muscle (light blue), IMAT (green), VAT (orange), and SAT (red).

AI generated body composition results were obtained from the lumbosacral region of each patient's preoperative CT scan. All CT scans were performed within 3 months of surgery and following any neoadjuvant therapy. Data on tissue volume (cm^3^) and average radiodensity (Hounsfield units, HU) for skeletal muscle (SM), intramuscular adipose tissue (IMAT), visceral adipose tissue (VAT), and subcutaneous adipose tissue (SAT) were collected with a pretrained and validated in‐house AI segmentation model [11, 12]. Three‐dimensional body composition for each tissue type was determined by assessing axial CT scan slices within the lumbosacral region as determined by a trained investigator (A.B. and J.Y.). Tissue volume was determined by multiplying the area of tissue present in each axial slice by the CT slice thickness. Average tissue radiodensity (HU) was determined by the sum of the mean radiodensity in each axial slice, for each tissue compartment, divided by the number of slices in the lumbosacral region. Figure 1 demonstrates a single axial image of a CT scan used for body composition analysis, the four tissue types of interest (SM, IMAT, VAT, and SAT) have been color coded based on the AI algorithm analysis.

We conducted our body composition analysis by categorizing patients into quartiles and comparing patients at greatest risk (lowest SM volume quartile or greatest IMAT/VAT/SAT volume quartile) to all remaining patients. A previous 2D body composition study in our patient cohort found that 62.5% of patients had sarcopenia [15]. Based on this result, we felt that the worst body composition quartile would represent the highest risk patients within our cohort.

### 2.3 Statistical Analysis

Descriptive statistics were used for baseline patient demographics to compare between groups. For continuous variables, median values and interquartile range (IQR) were compared using the Mann–Whitney *U* test, whereas categorical values were assessed using Fisher's exact test. All statistics testing utilized two‐tailed test results. Body composition analysis was conducted by comparing sex specific quartiles as no preexisting 3D body composition values have been validated.

## 3 Results

**FIGURE 2:**
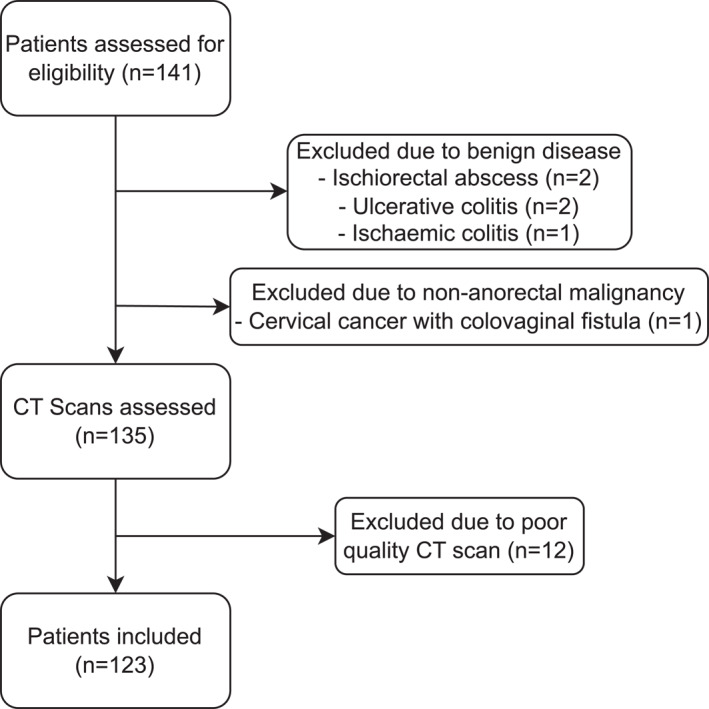
Patient inclusion/exclusion flow diagram.

A total of 123 patients who underwent APR for anorectal malignancy at WH were included in our study. Figure 2 outlines the exclusion reasons for an additional 18 patients that underwent an APR during the study period; 5 due to benign disease, 1 due to nonanorectal malignancy, and 12 because of poor quality CT scans. Eighty‐seven (71%) patients were male and the median age at surgery was 63.4 (IQR 54.7–73.8). Primary closure was performed for 72 (59%) patients whereas an IGAM flap was performed in 51 (41%) patients. Patient demographics can be seen in Table 1 along with the summary statistics for the IGAM and PC cohorts. There was a greater percentage of female patients in the IGAM group compared with the PC group (39% vs. 22% and *p* = 0.045). All patients undergoing salvage APR for anal SCC required an IGAM flap; therefore, a difference in the histological indication for surgery between groups was seen (*p* = 0.002). The incidence of type 2 diabetes was equivalent between groups (21.6% IGAM vs. 26.4% PC and *p* = 0.806) with a single patient in the PC group having type 1 diabetes. TNT was administered to 14 (11.4%) of patients in this study, 12 receiving induction chemotherapy, and two consolidation chemotherapy. There was no statistical difference in postoperative outcomes between patients receiving TNT an those who did not (wound infection *p* = 0.460, wound dehiscence *p* = 0.734, and return to theater *p* = 0.297).

**TABLE 1:** Patient demographic data.

	Entire cohort	Perineal closure method		*p*‐value
	*n* = 123	IGAM^a^	PC^b^	
		*n* = 51	*n* = 72	
Sex				0.045
Male	87 (71%)	31 (61%)	56 (78%)	
Female	36 (29%)	20 (39%)	16 (22%)	
Age (years), (median, IQR)	63.4 (54.7–73.8)	61.8 (51.8–75.0)	63.9 (55.0–73.2)	0.617
Body mass index	26.9 (23.4–30.0)	26.0 (23.4–30.0)	27.0 (23.5–30.0)	0.421
Smoking status				
Never	52 (42.3%)	21 (41.2%)	31 (43.1%)	0.843
Ex‐smoker	43 (35.0%)	17 (33.3%)	26 (36.1%)
Current	28 (22.7%)	13 (25.5%)	15 (20.8%)
Diabetic status				
Non‐diabetic	92 (74.8%)	40 (78.4%)	52 (72.2%)	0.806
Type I	1 (0.8%)	0 (0%)	1 (1.4%)
Type II	30 (24.4%)	11 (21.6%)	19 (26.4%)
Type of APR^c^				
Open	75 (61.0%)	30 (58.8%)	45 (62.5%)	0.852
Laparoscopic/lap assisted	48 (39.0%)	21 (41.2%)	27 (37.5%)
Histology				
Adenocarcinoma	113 (91.9%)	43 (84.3%)	70 (97.2%)	0.002
Mucinous	3 (2.4%)	1 (2.0%)	2 (2.8%)
Squamous cell carcinoma	7 (5.7%)	7 (13.7%)	0
Operative duration (min)	360 (233–503)	524 (451–600)	249 (210–337)	< 0.001
Length of stay (days)	10 (8–14)	14 (10–18)	9 (7–11)	< 0.001
Length of follow‐up (months)	26.1 (10.8–44.7)	28.3 (11.7–54.1)	21.2 (10.7–37.2)	0.150
Flap loss	2 (1.6%)	2 (3.9%)	N/A	
Wound infection	26 (21.1%)	13 (25.5%)	13 (18.1%)	0.371
Wound dehiscence	26 (21.1%)	12 (23.5%)	14 (19.4%)	0.655
Return to theater		15 (29.4%)	7 (9.7%)	0.008

^a^ Inferior gluteal artery myocutaneous.

^b^ Primary closure.

^c^ Abdominoperineal resection.

Sex specific body composition outcomes (volume, volume to height ratio, and radiodensity) for each tissue type (SM, IMAT, VAT, and SAT) are listed in Table S1. There were no baseline body composition differences between IGAM and PC groups for either male or female patients except for a greater SM‐to‐IMAT volume ratio (12.6 vs. 9.7, *p* = 0.039) for female patients.

Male patients undergoing IGAM reconstruction with greater IMAT volume were more likely to develop a wound dehiscence (40 vs. 4.8% and *p* = 0.027). Similarly, increased infection rates were seen in this cohort who had a lower SM‐to‐IMAT volume ratio (60 vs. 19% and *p* = 0.04). These findings were not replicated for female patients. Tables S2 and S3 show the impact of body composition on surgical complications (wound infection, wound dehiscence, and return to theater) for IGAM and PC patients. There was no significant impact of known risk factors (age, BMI, ECOG, smoking, and diabetic status) with the occurrence of wound infection, dehiscence, or return to theater; a multivariate analysis of confounding factors can be seen in Table S4.

Return to theater was more common for IGAM patients (29.4% vs. 9.6%, *p* = 0.008); however, there was no difference in wound infection or wound dehiscence rates based on method of perineal closure. There was a longer operative duration (minutes) in the IGAM group compared to the PC group (524, 451–600 vs. 249, 210–337, and *p* < 0.001). This was also seen in hospital length of stay (LOS) with a significantly greater number of days spent in hospital for the IGAM group (14, 10–18 vs. 9, 7–11, and *p* < 0.001).

## 4 Discussion

Myosteatosis is associated with worse overall survival in CRC treatment [16]. Recently, IMAT volume has been identified as a predictor of postoperative wound complications [17]. Our study showed increased rates of perineal wound dehiscence in male patients with greater IMAT volume (40% IGAM vs. 4.8% PC and *p* = 0.027) who underwent IGAM flap reconstruction. Similarly, in this cohort, those who had a lower SM‐to‐IMAT volume ratio (60% vs. 19% and *p* = 0.04) had a greater incidence of wound infection. We explored the relationship of SM‐to‐IMAT volume ratio to generate a SM index that can provide a holistic single value to indicate SM volume and quality. Lower values indicate greater adipose deposition within skeletal muscle which is evidence of myosteatosis and has been shown to reduce strength and skeletal muscle function [18]. This index has provided useful clinical information that necessitates further research. Malnutrition, obesity, older age, and a sedentary lifestyle all result in an increased IMAT volume and are shared risk factors for postoperative wound complications [19, 20]. Myosteatosis and high IMAT volume are radiological signs of poor nutrition and are associated with adverse postoperative outcomes in colorectal surgery [17, 21]. These findings support the recent popularization of IMAT volume as a useful biomarker in CRC surgery.

We demonstrated SAT volume, when adjusted for patient height, was associated with a significant incidence in return to theater (RTT) for male patients undergoing IGAM flap reconstruction (62.5% vs. 17.4% and *p* = 0.027). This was predominately for wound dehiscence or flap failure. Despite having similar rates of wound dehiscence (23.5% IGAM vs. 19.4% PC and *p* = 0.655), patients who underwent PC were more likely to be managed nonoperatively (64% PC vs. 25% IGAM and *p* = 0.047), thereby avoiding RTT. This offers an explanation as to why SAT volume did not also predict RTT in male PC patients. Although the exact mechanism is not completely understood, increased subcutaneous adipose tissue is thought to cause poor wound healing as a result of vascular insufficiency, reduced collagen synthesis, and increased wound infection risk [22].

Within our institution, perineal wound closure is performed by either PC or IGAM flap reconstruction. The method of wound closure is decided by the treating clinician following a multidisciplinary team meeting based on patient characteristics and anorectal cancer pathology. Fifty‐one out of 123 (41%) patients included in our study had an IGAM flap performed. Baseline patient characteristics, including diabetes, smoking status, BMI, operative technique, and body composition between PC and IGAM patients, were comparable. Predictably, operative time and LOS were significantly longer for IGAM flap patients. At baseline, there are body composition differences between male and female patients. Males have greater lean body mass and VAT volume whereas female patients have increased SAT volume [23]. This, coupled with the significant difference (*p* = 0.045) in number of male and female patients between our IGAM (31 male and 20 female) and PC (57 male and 16 female) groups, meant we assessed the impact body composition has on postoperative outcomes for male and female patients separately.

Body composition results for female patients failed to identify significant clinical outcomes. This could be due to the effect of a small cohort size rather than a fundamental difference between male and female patients. Wound dehiscence following IGAM flap reconstruction occurred frequently (35%) in female patients compared to 16% for males; however, with only 20 patients in this cohort, the ability to detect a clinically significant impact of body composition on surgical outcomes is considerably reduced. Almasaudi et al. found similar results with male patients, exhibiting strong associations with body composition and postoperative wound outcomes following CRC surgery whereas no significant associated was noted for female patients despite 331 female patients included in their study [24]. Multiple hypotheses are offered to explain this sex difference including the adipose tissue distribution variation between male and female patients [24, 25]. Fuente‐Martín et al. showed that the metabolic function of adipose tissue varies between males and females; they identified that males have increased proinflammatory immune cells in subcutaneous adipose tissue with greater macrophage infiltration when compared to females which could also explain the variable predictive ability of body composition on wound outcomes [25]. Sex specific outcomes need to remain a focus of future research to better understand these differences.

The impact of sarcopenia on clinical outcomes has been increasingly studied over recent years. Unfavorable body composition subtypes, such as sarcopenia and sarcopenic obesity, have consistently been found to negatively impact rectal cancer outcomes. Meta‐analysis has shown sarcopenia to be an independent predictor of decreased overall survival, cancer recurrence, neoadjuvant therapy outcomes, and postoperative complications in rectal cancer treatment [26, 27]. Miller et al. showed increased postoperative wound infection incidence in sarcopenic patients following APR who had flap‐based reconstruction, although this was inclusive of both abdominal and perineal wounds [28]. When looking specifically at perineal complications, there was no difference between sarcopenic and nonsarcopenic patients [28]. Our study produced similar results in which SM volume and quality did not impact perineal wound complications for either PC or IGAM flap patients.

There is mixed evidence reported regarding the impact of visceral obesity on surgical outcomes. Zhou et al. found visceral obesity in combination with malnutrition to be associated with increased postoperative complication rates following rectal cancer resection [29]. Conversely, another study looking at laparoscopic rectal cancer resections found that VAT was not associated with the incidence of an anastomotic leak or 30‐day surgical complication rate [30]. In our cohort, VAT volume was not found to influence perineal wound outcomes or hospital LOS irrespective of sex or closure method.

To our knowledge, this is the first study utilizing AI derived 3D lumbosacral body composition assessment from preoperative CT scans to predict perineal wound outcomes following APR. We identified sex specific body composition parameters that predisposed patients to increased risk of perineal wound complications. Despite these findings, there were some limitations with our study. We acknowledge that the surgical indication for type of perineal closure was different, and therefore, the two patient groups compared had some underlying bias. The size of the perineal defect was also not prospectively measured, and we can only assume that the patients who required IGAM reconstruction had a larger perineal defect. The low number of female patients in this study has likely prevented the detection of clinically meaningful and significant results being observed, and therefore, further work is needed to truly understand how body composition influences female patients and their recovery following APR.

Identification and validation of lumbosacral tissue volumes that define specific body composition subtypes, such as sarcopenia, obesity, and sarcopenic obesity, are still required. Until these thresholds have been determined, analysis of 3D body composition data should continue by comparing quartiles. This method has been successfully used in our study and by Baastrup et al. who compared the effect of visceral obesity on rates of anastomotic leak and 30‐day complications following rectal cancer resections [30]. The 75th centile for VAT in the Baastrup et al. study (5.36 L, range 0.14–9.21 L) is comparable to our findings (5.14 L, range 0.05–12.50 L) [30]. A consistent method of analysis will allow for direct comparison between studies until specific 3D body composition thresholds have been validated.

It is unlikely that body composition based risk assessment would change the method of perineal closure performed as the underlying reason for requiring flap based reconstruction would be unchanged. However, risk stratification could help with early identification of patients at greater risk of perineal complications. This information could inform active intervention programs, including nutritional supplementation and prehabilitation, during the long neoadjuvant treatment period in the current era of total neoadjuvant therapy. This could augment an individual patient's risk profile with the aim of reducing rates of perineal wound complications and patient morbidity.

## 5 Conclusion

IMAT has been shown to be a promising predictor of wound infection and dehiscence in male patients undergoing IGAM flap reconstruction. Similarly, SM‐to‐IMAT volume ratio has been developed as a novel index and shows potential for predicting clinical outcomes, warranting further investigation. AI derived 3D lumbosacral body composition from pre‐operative CT scans is a useful tool in risk stratification of patients undergoing complex rectal cancer surgery and could help guide the use of intensive prehabilitation interventions.

## Author Contributions

**Alex Besson:** investigation, data curation, formal analysis, writing – original draft, writing – review and editing, visualization. **Ke Cao:** investigation, data curation, writing – review and editing, software. **Rory Kokelaar:** writing – review and editing. **Emina Hajdarevic:** investigation, writing – review and editing. **Lara Wirth:** investigation, writing – review and editing. **Josephine Yeung:** data curation, writing – review and editing. **Justin M. Yeung:** conceptualization, supervision, writing – original draft, writing – review and editing, project administration, methodology.

## Conflicts of Interest

The authors declare no conflicts of interest.

## Supporting information


Supporting Information S1

**Table S1**: 3D body composition data.

**Table S2**: Male patient surgical complications based on body composition.

**Table S3**: Female patient surgical complications based on body composition.

**Table S4**: Multivariate analysis of complication risk factors.

## Data Availability

The corresponding author has no research scholarship or funding to declare.
