# Supplementary material for: Artificial Intelligence‐Derived Intramuscular Adipose Tissue Assessment Predicts Perineal Wound Complications Following Abdominoperineal Resection
Source: World J Surg. 2025 Sep 15;49(11):3060–6. doi: 10.1002/wjs.70095 (PMC12582141; doi:10.1002/wjs.70095)
Supplement: Supplementary file 2 — Table S1: 3D body composition data. [file WJS-49-3060-s005.docx]

Supplementary Table I: 3D body composition data

|  | | IGAM^a^ flap | Primary Closure | P-value |
| --- | --- | --- | --- | --- |
| SM^b^ Volume (cm^3^) | Male | 7297.015 (5672.922 – 6302.651) | 6742.841 (5937.626 – 7684.033) | 0.408 |
|  | Female | 4496.371 (4157.631 – 5357.156) | 4225.891 (3921.798 – 4930.614) | 0.32 |
| SM Volume:Height (cm^3^:cm) | Male | 42.23975 (33.96959 – 46.86053) | 39.23773 (34.95014 – 44.37101) | 0.537 |
|  | Female | 28.12794 (26.74255 – 32.04662) | 26.35155 (25.00471 – 31.52541) | 0.236 |
| Muscle HU^c^ | Male | 39.1011 (33.76694 – 4659512) | 41.6525 (34.76776 – 46.31796) | 0.639 |
|  | Female | 40.39429 (35.70164 – 46.39802) | 38.36006 (30.28702 – 44.61153) | 0.262 |
| IMAT^d^ Volume (cm^3^) | Male | 468.0786 (263.8263 – 850.0485) | 407.9022 (338.3445 – 584.923) | 0.433 |
|  | Female | 349.7617 (261.4917 – 452.9903) | 443.3246 (325.3346 – 691.8615) | 0.14 |
| IMAT Volume:Height (cm^3^:cm) | Male | 2.631146 (1.48217 – 5.00285) | 2.45116 (1.86457 – 3.38113) | 0.572 |
|  | Female | 2.184294 (1.693053 – 2.766223) | 2.662737 (1.968837 – 4.731415) | 0.124 |
| IMAT HU | Male | -56.46046 (-61.60368 - -54.47879) | -56.08545 (-58.7069 - -54.71139) | 0.607 |
|  | Female | -57.0036 (-59.70121 - -54.87547) | -58.33312 (-64.93891 - -54.29474) | 0.519 |
| VAT^e^ Volume (cm^3^) | Male | 3388.841 (1070.034 – 5963.049) | 4000.073 (2683.993 – 5407.786) | 0.589 |
|  | Female | 2138.799 (1221.07 – 3301.292) | 2361.581 (1467.896 – 3250.66) | 0.741 |
| VAT Volume:Height (cm^3^:cm) | Male | 19.4761 (5.752874 – 35.69335) | 22.85756 (15.9359 – 31.77598) | 0.492 |
|  | Female | 13.61036 (7.442034 – 21.059) | 14.86018 (9.107331 – 21.55568) | 0.741 |
| VAT HU | Male | -90.68576 (-93.27948 - -7860658) | -8876213 (-93.47264 – 82.85864) | 0.993 |
|  | Female | -88.44895 (-92.67441 - -84.24645) | -84.43401 (-91.73613 - -81.81208) | 0.369 |
| SAT^f^ Volume (cm^3^) | Male | 4831.483 (3331.398 – 7132.001) | 4826.203 (3519.848 – 6954.162) | 0.998 |
|  | Female | 6704.509 (6150.865 – 8404.067) | 7131.513 (4213.705 – 10194.27) | 0.964 |
| SAT Volume:Height (cm^3^:cm) | Male | 26.84157 (18.71572 – 40.56008) | 28.28977 (20.81561 – 40.24598) | 0.917 |
|  | Female | 43.41572 (36.27996 – 53.21091) | 45.38903 (26.74952 – 65.40837) | 0.863 |
| SAT HU | Male | -95.51488 (-102.7174 - -88.03812) | -96.91009 (-101.3247 - -92.52933) | 0.761 |
|  | Female | -102.9946 (-105.8469 - -93.91941) | -99.6058 (-106.6237 - -93.01041) | 0.44 |
| SM:Total Fat Volume | Male | 0.7568048 (0.5677723 – 1.294542) | 0.6921067 (0.55403 – 0.9272886) | 0.513 |
|  | Female | 0.4596209 (0.3695426 – 0.6707361) | 0.4138781 (0.3137038 – 0.5925886) | 0.336 |
| SM:IMAT volume | Male | 15.44588 (9.0222127 – 27.43077) | 15.46558 (11.45163 – 21.64247) | 0.707 |
|  | Female | 12.66407 (9.598782 – 18.92428) | 9.66301 (6.032419 – 13.06474) | 0.039 |

a: Inferior Gluteal Artery Myocutaneous, b: Skeletal Muscle, c: Hounsfield Unit, d: Intramuscular Adipose Tissue, e: Visceral Adipose Tissue, f: Subcutaneous Adipose Tissue
