# Supplementary material for: Artificial Intelligence‐Derived Intramuscular Adipose Tissue Assessment Predicts Perineal Wound Complications Following Abdominoperineal Resection
Source: World J Surg. 2025 Sep 15;49(11):3060–6. doi: 10.1002/wjs.70095 (PMC12582141; doi:10.1002/wjs.70095)
Supplement: Supplementary file 3 — Table S2: Male patient surgical complications based on body composition. [file WJS-49-3060-s001.docx]

Supplementary Table II: Male patient surgical complications based on body composition

| Body composition | Outcome | IGAM^a^ | | P-value | Primary Closure | | P-value |
| --- | --- | --- | --- | --- | --- | --- | --- |
|  |  | Worse Quartile | |  | Worse Quartile | |  |
|  |  | Yes | No |  | Yes | No |  |
| SM^b^ volume | Wound Infection (%) | 22.2 | 36.4 | 0.677 | 15.4 | 20.5 | 1 |
|  | Wound Dehiscence (%) | 0 | 22.7 | 0.286 | 23.1 | 20.5 | 1 |
|  | Return to Theatre (%) | 22.2 | 31.8 | 0.689 | 7.7 | 11.4 | 1 |
| SM volume:Height | Wound Infection (%) | 33.3 | 31.8 | 1 | 7.7 | 22.7 | 0.426 |
|  | Wound Dehiscence (%) | 0 | 22.7 | 0.286 | 15.4 | 22.7 | 0.713 |
|  | Return to Theatre (%) | 22.2 | 31.8 | 0.689 | 7.7 | 11.4 | 1 |
| Muscle HU^c^ | Wound Infection (%) | 44.4 | 27.3 | 0.417 | 0 | 25 | 0.053 |
|  | Wound Dehiscence (%) | 22.2 | 15.8 | 0.613 | 15.4 | 22.7 | 0.713 |
|  | Return to Theatre (%) | 33.3 | 27.3 | 1 | 15.4 | 9.1 | 0.611 |
| IMAT^d^ volume | Wound Infection (%) | 50 | 23.8 | 0.222 | 0 | 24.4 | 0.097 |
|  | Wound Dehiscence (%) | 40 | 4.8 | 0.027 | 16.7 | 22.2 | 1 |
|  | Return to Theatre (%) | 21.1 | 23.8 | 0.417 | 16.7 | 8.9 | 0.596 |
| IMAT volume:Height | Wound Infection (%) | 55.6 | 22.7 | 0.105 | 0 | 25 | 0.053 |
|  | Wound Dehiscence (%) | 33.3 | 9.1 | 0.131 | 15.4 | 22.7 | 0.713 |
|  | Return to Theatre (%) | 44.4 | 22.7 | 0.385 | 15.4 | 9.1 | 0.611 |
| IMAT HU | Wound Infection (%) | 20 | 38.1 | 0.428 | 8.3 | 22.2 | 0.425 |
|  | Wound Dehiscence (%) | 20 | 14.3 | 1 | 8.3 | 24.4 | 0.427 |
|  | Return to Theatre (%) | 20 | 33.3 | 0.677 | 8.3 | 11.1 | 1 |
| VAT^e^ volume | Wound Infection (%) | 33.3 | 31.8 | 1 | 15.4 | 20.5 | 1 |
|  | Wound Dehiscence (%) | 11.1 | 18.2 | 1 | 15.4 | 22.7 | 0.713 |
|  | Return to Theatre (%) | 33.3 | 27.3 | 1 | 7.7 | 11.4 | 1 |
| VAT volume:Height | Wound Infection (%) | 33.3 | 31.8 | 1 | 15.4 | 20.5 | 1 |
|  | Wound Dehiscence (%) | 11.1 | 18.2 | 1 | 15.4 | 22.7 | 0.713 |
|  | Return to Theatre (%) | 33.3 | 27.3 | 1 | 0 | 13.6 | 0.319 |
| VAT HU | Wound Infection (%) | 14.3 | 37.5 | 0.379 | 26.7 | 16.7 | 0.455 |
|  | Wound Dehiscence (%) | 28.6 | 12.5 | 0.562 | 13.3 | 23.8 | 0.485 |
|  | Return to Theatre (%) | 28.6 | 29.2 | 1 | 6.7 | 11.6 | 1 |
| SAT^f^ volume | Wound Infection (%) | 62.5 | 21.7 | 0.074 | 14.3 | 20.9 | 0.714 |
|  | Wound Dehiscence (%) | 25 | 13 | 0.583 | 14.3 | 23.3 | 0.710 |
|  | Return to Theatre (%) | 50 | 21.7 | 0.185 | 14.3 | 9.3 | 0.629 |
| SAT volume:Height | Wound Infection (%) | 62.5 | 21.7 | 0.074 | 14.3 | 20.9 | 0.714 |
|  | Wound Dehiscence (%) | 37.5 | 8.7 | 0.093 | 14.3 | 23.3 | 0.71 |
|  | Return to Theatre (%) | 62.5 | 17.4 | 0.027 | 14.3 | 9.3 | 0.629 |
| SAT HU | Wound Infection (%) | 33.3 | 31.8 | 1 | 7.7 | 22.7 | 0.426 |
|  | Wound Dehiscence (%) | 11.1 | 18.2 | 1 | 0 | 27.3 | 0.05 |
|  | Return to Theatre (%) | 22.2 | 31.8 | 0.689 | 0 | 13.6 | 0.319 |
| SM:Total Fat Volume | Wound Infection (%) | 57.1 | 25 | 0.172 | 13.3 | 21.4 | 0.709 |
|  | Wound Dehiscence (%) | 14.3 | 16.7 | 1 | 13.3 | 23.8 | 0.485 |
|  | Return to Theatre (%) | 42.9 | 25 | 0.384 | 13.3 | 9.5 | 0.648 |
| SM:IMAT volume | Wound Infection (%) | 60 | 19 | 0.04 | 0 | 24.4 | 0.097 |
|  | Wound Dehiscence (%) | 30 | 9.5 | 0.296 | 25 | 20 | 0.702 |
|  | Return to Theatre (%) | 50 | 19 | 0.105 | 16.7 | 8.9 | 0.596 |

a: Inferior Gluteal Artery Myocutaneous, b: Skeletal Muscle, c: Hounsfield Unit, d: Intramuscular Adipose Tissue, e: Visceral Adipose Tissue, f: Subcutaneous Adipose Tissue
