# Supplementary material for: Artificial Intelligence‐Derived Intramuscular Adipose Tissue Assessment Predicts Perineal Wound Complications Following Abdominoperineal Resection
Source: World J Surg. 2025 Sep 15;49(11):3060–6. doi: 10.1002/wjs.70095 (PMC12582141; doi:10.1002/wjs.70095)
Supplement: Supplementary file 4 — Table S3: Female patient surgical complications based on body composition. [file WJS-49-3060-s002.docx]

Supplementary Table III: Female patient surgical complications based on body composition

| Body composition | Outcome | IGAM^a^ | | P-value | Primary Closure | | P-value |
| --- | --- | --- | --- | --- | --- | --- | --- |
|  |  | Worse Quartile | |  | Worse Quartile | |  |
|  |  | Yes | No |  | Yes | No |  |
| SM^b^ volume | Wound Infection (%) | 0 | 18.8 | 1 | 20 | 9.1 | 1 |
|  | Wound Dehiscence (%) | 0 | 43.8 | 0.249 | 0 | 18.2 | 1 |
|  | Return to Theatre (%) | 25 | 31.3 | 1 | 0 | 9.1 | 1 |
| SM volume:Height | Wound Infection (%) | 0 | 17.6 | 1 | 16.7 | 10 | 1 |
|  | Wound Dehiscence (%) | 0 | 41.2 | 0.521 | 16.7 | 10 | 1 |
|  | Return to Theatre (%) | 33.3 | 29.4 | 1 | 16.7 | 0 | 0.375 |
| Muscle HU^c^ | Wound Infection (%) | 0 | 18.8 | 1 | 40 | 0 | 0.083 |
|  | Wound Dehiscence (%) | 25 | 37.5 | 1 | 0 | 18.2 | 1 |
|  | Return to Theatre (%) | 25 | 31.3 | 1 | 0 | 9.1 | 1 |
| IMAT^\|\|^ volume | Wound Infection (%) | 0 | 18.8 | 1 | 40 | 0 | 0.083 |
|  | Wound Dehiscence (%) | 50 | 31.3 | 0.587 | 0 | 18.2 | 1 |
|  | Return to Theatre (%) | 50 | 25 | 0.549 | 0 | 9.1 | 1 |
| IMAT volume:Height | Wound Infection (%) | 0 | 17.6 | 1 | 33.3 | 0 | 0.125 |
|  | Wound Dehiscence (%) | 66.7 | 29.4 | 0.270 | 0 | 20 | 0.5 |
|  | Return to Theatre (%) | 66.7 | 23.5 | 0.202 | 0 | 10 | 1 |
| IMAT HU | Wound Infection (%) | 0 | 17.6 | 1 | 33.3 | 0 | 0.125 |
|  | Wound Dehiscence (%) | 33.3 | 35.3 | 1 | 0 | 20 | 0.5 |
|  | Return to Theatre (%) | 33.3 | 29.4 | 1 | 0 | 10 | 1 |
| VAT^e^ volume | Wound Infection (%) | 0 | 20 | 0.539 | 25 | 8.3 | 0.45 |
|  | Wound Dehiscence (%) | 40 | 33.3 | 1 | 0 | 16.7 | 1 |
|  | Return to Theatre (%) | 40 | 26.7 | 0.613 | 0 | 8.3 | 1 |
| VAT volume:Height | Wound Infection (%) | 0 | 20 | 0.539 | 25 | 8.3 | 0.45 |
|  | Wound Dehiscence (%) | 40 | 33.3 | 1 | 0 | 16.7 | 1 |
|  | Return to Theatre (%) | 40 | 26.7 | 0.613 | 0 | 8.3 | 1 |
| VAT HU | Wound Infection (%) | 16.7 | 14.3 | 1 | 33.3 | 7.7 | 0.35 |
|  | Wound Dehiscence (%) | 33.3 | 35.7 | 1 | 0 | 15.4 | 1 |
|  | Return to Theatre (%) | 33.3 | 28.6 | 1 | 0 | 7.7 | 1 |
| SAT^f^ volume | Wound Infection (%) | 0 | 18.8 | 1 | 20 | 9.1 | 1 |
|  | Wound Dehiscence (%) | 50 | 31.3 | 0.587 | 0 | 18.2 | 1 |
|  | Return to Theatre (%) | 25 | 31.3 | 1 | 0 | 9.1 | 1 |
| SAT volume:Height | Wound Infection (%) | 0 | 18.8 | 1 | 20 | 9.1 | 1 |
|  | Wound Dehiscence (%) | 50 | 31.3 | 0.587 | 0 | 18.2 | 1 |
|  | Return to Theatre (%) | 25 | 31.3 | 1 | 0 | 9.1 | 1 |
| SAT HU | Wound Infection (%) | 20 | 6.7 | 1 | 25 | 8.3 | 0.45 |
|  | Wound Dehiscence (%) | 80 | 20 | 0.031 | 0 | 16.7 | 1 |
|  | Return to Theatre (%) | 40 | 26.7 | 0.613 | 0 | 8.3 | 1 |
| SM:Total Fat Volume | Wound Infection (%) | 0 | 18.8 | 1 | 40 | 0 | 0.083 |
|  | Wound Dehiscence (%) | 25 | 37.5 | 1 | 0 | 18.2 | 1 |
|  | Return to Theatre (%) | 0 | 37.5 | 0.267 | 0 | 9.1 | 1 |
| SM:IMAT volume | Wound Infection (%) | 0 | 16.7 | 1 | 28.6 | 0 | 0.175 |
|  | Wound Dehiscence (%) | 50 | 33.3 | 1 | 0 | 11.1 | 0.475 |
|  | Return to Theatre (%) | 50 | 27.8 | 0.521 | 0 | 11.1 | 1 |

a: Inferior Gluteal Artery Myocutaneous, b: Skeletal Muscle, c: Hounsfield Unit, d: Intramuscular Adipose Tissue, e: Visceral Adipose Tissue, f: Subcutaneous Adipose Tissue
