# Supplementary material for: Artificial Intelligence‐Derived Intramuscular Adipose Tissue Assessment Predicts Perineal Wound Complications Following Abdominoperineal Resection
Source: World J Surg. 2025 Sep 15;49(11):3060–6. doi: 10.1002/wjs.70095 (PMC12582141; doi:10.1002/wjs.70095)
Supplement: Supplementary file 5 — Table S4: Multivariate analysis of complication risk factors. [file WJS-49-3060-s003.docx]

Supplementary Table IV: Multivariate analysis of complication risk factors

| Variables | Odds Ratio (95% CI^a^) | p-value |
| --- | --- | --- |
| Wound infection   - Age - BMI^b^ - Smoking status - Diabetic status - ECOG^c^ | 0.98 (0.95-1.02)  1.02 (0.93-1.11)  1.38 (0.76-2.50)  1.48 (0.89-2.46)  0.97 (0.34-2.78) | 0.300  0.693  0.283  0.127  0.959 |
| Wound Dehiscence   - Age - BMI - Smoking status - Diabetic status - ECOG | 0.98 (0.95-1.02)  1.04 (0.95-1.13)  1.67 (0.91-3.07)  1.42 (0.85-2.38)  0.97 (0.34-2.78) | 0.265  0.384  0.096  0.181  0.978 |
| Return to theatre   - Age - BMI - Smoking status - Diabetic status - ECOG | 1.00 (0.96-1.04)  1.02 (0.94-1.12)  1.32 (0.69-2.53)  1.54 (0.92-2.59)  1.41 (0.52-3.82) | 0.929  0.614  0.391  0.103  0.500 |

a: Confidence Interval, b: Body Mass Index, c: East Cooperative Oncology Group
